# Risk Awareness and Impediments to Disaster Preparedness of Foreign Residents in the Tokyo Metropolitan Area, Japan

**DOI:** 10.3390/ijerph191811469

**Published:** 2022-09-12

**Authors:** Bismark Adu-Gyamfi, Rajib Shaw

**Affiliations:** Graduate School of Media and Governance, Keio University (SFC), 5322 Endo, Fujisawa-shi 252-0882, Japan

**Keywords:** disaster preparedness, risk awareness, Extended Parallel Process Model, Tokyo Metropolitan area, foreign residents

## Abstract

The likelihood of a mega-earthquake, the COVID-19 pandemic, and the threat of other disasters in the Tokyo Metropolitan area have necessitated collective responsibilities to take all possible actions to reduce their impacts. The experiences from past disasters have, however, highlighted the plight of foreign residents in coping with disasters and have reinvigorated calls for rigorous counteractions. As the population of foreign residents continues to increase in the metropolis, this research examines their awareness of risks and the obstacles that hinder disaster preparedness in the wake of future disasters. An Extended Parallel Process Model is utilized to analyze both secondary and primary data sources, and the results reveal that 65% perceive the severity of disaster as a threat and believe in a likelihood of occurrence in the city; however, the confidence to undertake some recommended countermeasures are lacking (with low efficacy of 70%). This is causing many to control their fear by not actively seeking further risk information or participating in disaster prevention activities. In consideration of these instances, the study recommends a collective approach built upon the merits of stakeholder engagements to provide vicarious experiences, verbal persuasions, and mastery experiences to boost the response capacities of foreign residents for disaster preparedness. This will be relevant for city authorities to enhance risk communication and foster foreigner–Japanese community integration.

## 1. Introduction

The change in nature and characteristics of disasters in recent years have equally necessitated robust and dynamic approaches viewed as countermeasures to these phenomena. The approaches encompass a broad understanding of hazards, risks, vulnerabilities, and coping mechanisms [1] which are generally addressed under a broader scope of disaster preparedness [2]. Disaster preparedness, in this context, refers to “the knowledge and capacities developed by governments, response and recovery organizations, communities and individuals to effectively anticipate, respond to and recover from the impacts of likely, imminent or current disasters” [2]. This content makes references to several stakeholders and other activities which must all be synchronized under the preparedness scope. Such activities range from a micro-level individual effort to rather macro-level collective countermeasures which are vital components of the Sendai Framework for Disaster Risk Reduction 2015–2030 (SFDRR). Per the guiding principles of the SFDRR, collective stakeholder actions and cooperation in risk reductions and disaster preparedness are key factors to substantially reduce the impacts of disasters [3]. The process of synchronizing efforts for a collective disaster preparedness approach is highly incorporated in the public help (kojo), mutual help (kyojo), and self-help (jijo) strategy adopted by the Japanese government after the 6.9 magnitude Great Hanshin-Awaji Earthquake in 1995 [4]. It hinges on the premise of an obligation by the central and local governments to provide the needed resources and capabilities to aid the prediction, analysis, and anticipation of hazards and risks, as well as make all efforts to assist the populace in preparing before, during, and after disasters. Per such actions, communities and residents also have the responsibility to “…take their own measures to prepare for disaster and by participating in voluntary disaster prevention groups, etc.*”* as further enshrined in the Japanese Disaster Countermeasures Basic Act (Act No. 223, 15 November 1961) Article 7.2. 

This overall process places the final decision on disaster preparedness measures within the hands of the individual such that, the ability to reduce or prevent the impact of a disaster depends on the level of responsibility or compliance with recommended countermeasure actions [5,6]. Although empirical evidence suggests that Japanese people are generally prepared for disasters [7], foreign residents in the country are assumed to be the opposite [8]. Reasons attributed to the latter include cultural differences, lack of participation in local disaster measures, risk information and communication challenges, countries of origin, and others that inhibit them from knowing what actions to take toward disaster preparedness [9,10,11]. Further studies have, however, explored other characteristics of disaster preparedness of foreign residents in the country and provide counter-arguments to these attributions by suggesting that, some foreign residents rather show disaster preparedness attributes similar to Japanese nationals in disaster preparedness activities [12,13], implying that, hindrances to disaster preparedness knowledge may not be the major issue. Therefore, if foreign residents show some signs similar to Japanese nationals, and have some knowledge of disaster preparedness, then what other reasons could be attributed to the description of the vulnerability and limited disaster preparedness of foreign residents in the country? This study looks into these issues from a psychological perspective where the decision to undertake certain actions stem from some intrinsic decision-making process. For this process, the extended parallel process model (EPPM) is adopted as a tool to understand the perspective behaviors of foreign residents in the Tokyo Metropolitan area.

The paper is organized into six sections, with the first and second giving an overview and the scope of the study. The third provides a theoretical background and places it in the context of the current status quo of disaster preparedness in the metropolitan area. The rest of the sections consider a survey undertaken, its results, and a discussion of the results in perspectives. It then concludes with the major findings, limitations, and future works.

## 2. Foreign Residents in the Tokyo Metropolitan Area

Data from the Tokyo Metropolitan Government estimate the population of foreign residents in the city to be 577,329, with approximately 3.1% of the total population in the metropolis, which is an increase from 2.6% in 2000 [13]; however, comparing the population figure of 112,523 in 1980 suggests a more than 200% increase in the population number and this represents an average annual growth rate of 4%. The changes also correspond with changes and amendments in the immigration policies of Japan, such as the establishment of a Technical Intern Training Program in 1993 and a revision to Japan’s Immigration Control Act in 2009 [14]. These notwithstanding, the population saw a slight decline from the year 2020 as a result of the COVID-19 pandemic which caused the closure of all entry ports to Japan and a subsequent barring of non-Japanese nationals from entering the country as part of the pandemic countermeasures. However, there are indications that the population of foreigners will start to rise once the borders of the country are fully opened or relaxed. Looking at the rising number of foreign residents, it is rather occurring at a time when the Tokyo Metropolitan Government envisages a mega-earthquake to potentially hit the city at some time in the future. Per the various scenarios, building damage has been estimated, as of 2012, to be 443,800 (with 304,300 at the Tokyo Bay and 139,500 in the Tama area). Casualties were also estimated to be 14,400 deaths and 248,700 injuries [15]. Furthermore, the capital city has equally witnessed the highest number of COVID-19 infections and deaths across the entire country. These have necessitated the calls to take all actions to reduce the impacts of both current and future risks or disasters by taking full participation in risk management activities for which foreign residents play a key part in the casualty reduction. Previous studies have pointed out that the distribution patterns of foreign residents in the city depict scenarios where many foreign residents are mostly located in the eastern portions of the metropolis and are further concentrated within a 10 km radius of the Minato city area, which is already prone to many disasters. Moreover, within such enclaves in the metropolis, there is the Indian community mostly found in the Koto and Edogawa cities, the Korean community in the Shinjuku area, and the Chinese community mostly found in the Itabashi city. These bring an element of both homogeneity and heterogeneity among the foreign residents’ population in the metropolis and may offer both advantages and disadvantages to disaster management planning in the metropolitan area [16]. 

## 3. Literature Review

### 3.1. The Extended Parallel Process Model (EPPM) and Disaster Preparedness Behaviors

Humans face numerous threats and risks that either affects the individual as a person or his/her environment. Some of these threats or risks are unavoidable or inevitable but for those that can be avoided, several approaches are undertaken to prevent the threat from occurring, manage the threat, or find solutions for minimizing the impact, especially when it affects the lives of people [16,17]. One effective way to initiate such avoidable solutions resides in the effectiveness of the risk communication components, which include the description of the characteristics of risks, the preventative measures, and some level of self-target information mainly customized to an intended recipient [18]. In such situations, such an approach has been adopted with the intention of changing behaviors or attitudes toward certain risks [19]. This scenario has been highly utilized in the healthcare sector to persuade changes in the attitudes toward certain diseases, as well as to discourage some behaviors that jeopardize the health conditions of people and are undertaken within the context of behavioral modeling. These include the Protection Motivation Theory [20], the Health Belief Model [21], as well as the Extended Parallel Process Model [22]. These are sometimes referred to as “fear theories” due to the incorporation of a fear factor as a core element of the risk communication process [23]. 

Although these models are implemented in similar fashions, the Extended Parallel Process Model (EPPM) is widely used in behavioral change simulations due to its incorporation of the fear factors in its theoretical assumptions [24]; hence, it has been adopted to understand the fear factor of the COVID-19 pandemic threat, the fear factor associated with the risk of cardiovascular diseases due to tobacco smoking [23], the fear of contracting influenza from working in hazardous conditions [25], and the fear of developing colorectal cancer as a method of enhancing cancer screening [26]. Per this model, in order to initiate actions towards unforeseen dangers, risk communication should consist of information composed of two elements such that, one portrays a fear factor in a bid to convince the recipients of the intensity of the threat (threat component), while the other places convincing countermeasure approaches or interventions to instigate confidence (efficacy component) [25]. Therefore, per the realization of the model, both the threat and efficacy elements of the message must be duly accepted by the receivers to achieve the required action. At this stage, the dangers associated with the threat could be controlled per the initiated actions; however, in an event where the threat element of the information is not accepted, then the entire message is ignored. Furthermore, if the threat component is accepted but the confidence for the efficacy is not, then the threat message activates fears, which in turn prompts the receivers to devote their efforts to managing the fear; ultimately, rejecting the entire message as a whole. This stage is referred to as “fear control” [16,19]. 

All these have been successful in identifying the position and level of attitudes toward threats through the fear it portrays. This process has, in recent years, extended to disaster management frontiers for finding clues to the behaviors of people towards disaster preparedness. It hinges on disaster risk awareness and the transition of such knowledge into the motivations to seek countermeasures [27]. For instance, a past study adopted the EPPM to model the ”preparedness behaviors of university employees” against disaster preparedness and it was able to associate trigger behaviors for disaster preparedness [28] and showed great prospects of being used in other disaster preparedness behaviors. 

### 3.2. Portraying the Fear Factor for Disaster Preparedness in the Tokyo Metropolitan Area

**Figure 1:**
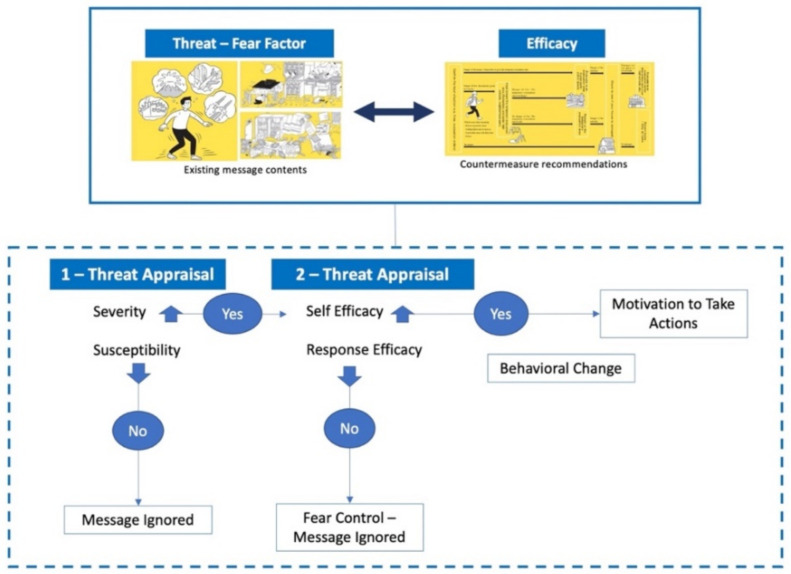
A framework of EPPM in the Tokyo Metropolitan Area.

Contextualizing the EPPM in the case of the Tokyo Metropolitan area can generally be looked at as the contents of risk communication in the form of information delivery to residents in the city and most especially foreign residents. Per existing study findings [29], various methods exist for risk communication and the portrayal of a fear factor in that regard can be established by virtual and physical risk communications methods. Scouting through disaster prevention information from the Tokyo Government’s websites provides sections on the risks and hazards in the city, disaster effects and impacts, warnings, and the efforts to prevent and protect oneself should any emergency occur [30]. However, printed copies of the disaster prevention guidelines in the city provide an alternative to risk communication in the city which, in turn, serves as the physical component of the process. Although many versions of such books exist, the “*TOKYO BOSAI*” booklet is said to be the most popular and ambitious project aimed at disaster risk awareness and preparedness. This project distributed the disaster awareness and preparedness booklet to more than 6 million households in the city with over 8 million copies printed [31]. The content of the *TOKYO BOSAI* guidebook can be referenced to consist of both threats and efficacies with the beginning pages stating the question, “it is predicted that there is a 70 percent possibility of an earthquake directly hitting Tokyo within the next 30 years. Are you prepared?”. This in its entirety can create fear for people to take action to avert its impacts. Furthermore, part two of the book highlights the relevance of the actions deemed vital to prevent disaster impacts which, in reference to the EPPM model, could be termed within the scope of efficacies. In summary of the situation in risk communication by authorities, Figure 1 illustrates the framework of the EPPM model in reference to the Tokyo Metropolitan area. 

## 4. Approach and Methodology

The approach for this study followed a set of coordinated activities to elucidate the perceived reactions to disasters as well as related activities and was designed in three parts. It began with a questionnaire survey that targeted any foreign resident living within the Tokyo Metropolitan area irrespective of their nationality. The first part brought out the respondents’ experiences with disasters and other risks in the metropolis. The disaster or risk-related experiences designed for this study shared similar traits with other studies [28]. These experiences were solicited from the survey in two dimensions. The first looked at the experience through contact with the hazard, while the other looked at the impact as a result of such encounters. The results from these experiences were measured and compared with the supposed response capacities of the respondents and how they perceived them. This was the second part of the process and was categorized under perceived threats and perceived susceptibility to the threats. The last part of the process then combined all the elements of the responses to analyze the overall efficacies of the threats, which were examined under perceived self-efficacy and response efficacy. 

### 4.1. Survey Instrument

A questionnaire comprising of questions grouped under various themes was designed using the Survey Monkey online platform and was intended for any foreign resident living in the metropolis. To ensure that only foreign residents responded to the questions, an introductory note was given before the commencement of the questionnaire to explain the rationale and the target population. Additionally, questions about their country/area of origin and visa status were used to screen any responses by native Japanese. The questionnaire covered questions about the demographic characteristics of responses and experiences with other sets of disaster-related activities. A link and QR codes of the questionnaire were then shared on identified foreign resident groups in Tokyo on Facebook, the LINE app, and other internet platforms. The collection of responses started on 12 April and ended on 5 June 2022, due to dwindling responses. In all, 107 responses were gathered. 

### 4.2. Part One

#### 4.2.1. Disaster Experience 

Adopting and modifying the definition of disaster by the UNISDR 2009, disaster experience in this study was defined to mean a respondent witnessing “a serious disruption of the functioning of a community” due to the sudden occurrence of a hazard. Empirical studies have given four types of disaster experiences including direct and indirect disaster experiences, as well as vicarious and life experiences [32]. These manifest in different aspects; therefore, the responses to this experience were collected using the following question:

1. Have you experienced/witnessed the following hazards/disasters in the Tokyo Metropolitan area before?

Options: earthquakes, flooding, tsunamis, severe thunderstorms, landslides/mudslides, severe cold/hot weather, disease pandemic, other disasters, or none of the above. 

The responses were coded with “1” if the answer to each question was “yes” and coded “0” if the answer was “no”. The “none of the above” responses were also coded “0”. The total disaster experience was calculated for each respondent by summing the scores of all answers. 

#### 4.2.2. Impact of Disasters

Disaster impacts occur in many forms and affect people in different dimensions, but the experience of each effect offers a clue as to the range of impact and to the potential approach to adapt. Adopting further the description of disaster impacts from UNISDR 2009, the impacts of disasters in this study looked at how much of that experience had been felt in reference to a respondent witnessing a loss of relative/acquaintance(s), incurred injuries, and “other negative effects on their physical, social and mental wellbeing, or damage to properties” due to a disaster. For this, the following questions were asked:

Could you describe your experience with the impact of a disaster/disease pandemic in the Tokyo Metropolitan area?

1. Saw others injured/killed, was injured myself, could not contact my family, I was ordered to leave my home/neighborhood, I had to temporarily quit school/work, I was confined indoors for a long time, I could not get access to essential utilities (e.g., water and electricity).
2. Saw a family/friend/colleague/close associate infected/died from an infectious disease, other, or none of the above.

Similar to the earlier approach, the responses were coded with “1” if the answer to each question was “yes” and coded “0” if the answer was “no”. The “none of the above” responses were also coded “0”. The total disaster impact was calculated for each respondent by summing the scores of all answers.

### 4.3. Part Two

#### 4.3.1. Perceived Threats

This was measured from the message and portrayal of disasters/hazards as threats to lives and properties; therefore, the respondents were asked if they believed the following:

1. Earthquakes, Tsunamis, and other disasters can cause severe damage or loss of lives.
2. COVID-19 and other infectious diseases can cause severe illness or death.

A “yes” answer to each was coded as “1” while the “no” responses were coded as “0”. The total perceived threat was then calculated by summing the responses to the two questions. The results gave a range from minimum to maximum in terms of the total score for each respondent. A mean value for the total scores was calculated and based on this, and scores below the mean value were termed as a “low perceived threat” while values above the mean were classified as a “high threat”. This process has been used by many researchers as a way to convert continuous numbers to dichotomous values [28].

#### 4.3.2. Perceived Susceptibility

Perceived susceptibility here refers to evaluating the respondents’ perception of the likelihood of a threat in the context of the definition quoted by [24].

Hence, per this study, the questions to solicit for this were based on the following variables:

1. How likely do you think a severe earthquake, flood, mudslide/landslide, typhoon, and other disasters could occur in your area of residence? (Each variable was asked as an independent question).
2. How likely do you think you could contract COVID-19 in Tokyo?

Using a 5-point Likert scale, the respondents were asked about their perception of the likelihood of such events by using (high) 5–very likely to (low) 1–very unlikely. Referencing the recommended method of analyzing Likert-type data and scale (Boone, Jr 2012), the frequencies, median, and mode of each variable were calculated to understand the distribution of each variable. To further categorize the responses to ascertain the level of perceived susceptibility, the mean score for all the variables representing the perception of the likelihood of the events (variables) was calculated for each respondent. This represented the total likelihood of the events; therefore, a mean value was calculated with values lower than the mean representing a low perceived susceptibility. Values higher than the mean, on the other hand, were referenced as a high perceived susceptibility.

### 4.4. Part Three

#### 4.4.1. Self-Efficacy

As stated in the above sections, the disaster and hazard prevention protocols of Japan require residents to take various actions to prevent or reduce the impact of disasters. This part of the questionnaire sought to inquire about the abilities of respondents in taking action. As such, the following questions were asked:

1. How confident are you that you will know what to do should any disaster occur?
2. How confident are you that you will be able to assist/direct others on what to do should any disaster occur (evacuation and directions, etc.)?
3. How confident are you that you are avoiding the 3Cs of COVID-19 infections (e.g., closed spaces, crowded spaces, close-contact settings)?

For each of the questions, the respondents were to select from a 5-point Likert scale of “not confident at all” representing 1 (low) to “very confident” representing 5 (high). The responses were converted to low/high self-efficacies using a process similar to the one conducted for perceived susceptibility.

#### 4.4.2. Response Efficacy

Response efficacy asserts that, the belief in a recommended behavior works in yielding certain outcomes [33]. Previous studies on the matter have usually asked questions that directly relate to a recommended action toward certain events. Such responses often generate positive results in the sense that people might not give answers opposite to the recommended actions because no alternative actions are proposed. For instance, earlier studies [24] assessed the response efficacies of recommended actions for COVID-19 prevention in the Republic of Korea using questions such as, “how helpful do you think the following actions are in preventing the spread of COVID-19?”, for which outlined actions such as wearing a mask, washing hands, cough etiquette, etc., were provided. Under no alternative recommendations to such measures, responses are bound to sway positively; hence, responses from the study were nearly 80% affirmative from the respondents. Similar studies share similar characteristics [23]; however, for this study, the assumption was that if a person believed in the recommended actions, there would be a level of perseverance to seek information related to such activities. Therefore, the information on response efficacy was solicited with questions that sought to inquire how often they would seek information for recommended actions toward disaster prevention or risk reduction. For this, the following questions were asked:

1. How often do you read the information on disasters from the city hall/neighborhood notice board/other public notice boards?
2. How often do you read the information on disasters from your school/workplace?
3. How often do you search for information on disasters/infectious diseases on the internet?
4. How often do you participate in disaster/evacuation drills at your workplace/school/local community?

This was a 4-point Likert scale that prompted the respondents to select from “not at all” representing 1 (low) to very often, representing 4 (high). Again, the frequencies, modes, and means were calculated for all responses and the total was converted to high/low response efficacies using the mean score as the splitting point. 

### 4.5. Statistical Analysis

The data were analyzed using SPSS software (https://www.ibm.com/products/spss-statistics, accessed on 6 July 2022) and a normality test was conducted for the disaster-related variables to ascertain the skewness and kurtosis values of the data. Reliability tests were also conducted for the impact of disasters, perceived threat, perceived susceptibility, self-efficacy, and response efficacy variables.

## 5. Results

### 5.1. Demographic and Other Characteristics of Respondents 

**Figure 2:**
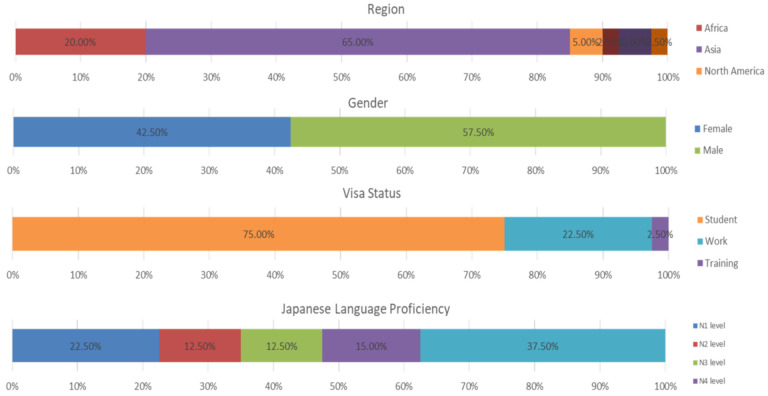
Demographic characteristics.

The demographic characteristics included the places of origin of the respondents and, as depicted in Figure 2, a greater portion of the respondents came from the Asian Pacific, with a percentage rate of 56, while others originated from Africa, Europe, North/South America, and Oceania. The figure again gives a glimpse of the gender composition of the respondents with more than 50% being male. In terms of their visa statuses, more than 75% hold student visas and with more than 50% within that group having lived in Tokyo for less than 5 years. Despite this, the majority of the respondents, representing nearly 80%, have lived in the metropolitan area for 1 to 5 years. The figure gain shows the level of Japanese language proficiency among the respondents and, as depicted, the majority are in the “no or limited Japanese language proficiency in the N5/N4” category. In terms of the distributions among the various characteristics, about 60% of people with student visas have low levels (thus, N5/N4 levels), while in the working group, more than 60% of its respondents possess high proficiency levels from the N3 to N1 levels. Referencing the other variables also showed that the respondents who have lived in the country as a whole for more than 5 years have a better command of the Japanese language, as many are found within the N3, N2, and N1 cohorts. This is in line with other assertions that, as people live in the country for a long time, their level of Japanese language proficiency also improves. 

### 5.2. Perception of Hazards and Disasters

**Figure 3:**
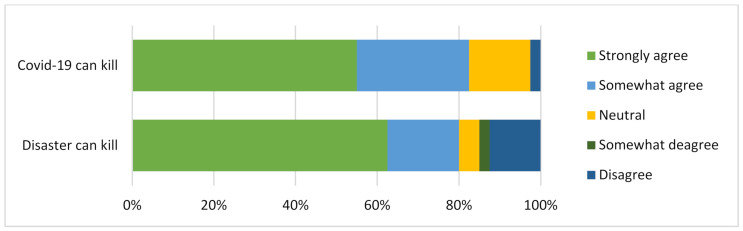
Perception of disaster/hazard impact.

One key element of disaster preparedness begins with the realization that there is the presence of hazards or risks as well as a possible impact should disasters occur. This leads to the general understanding of whether a person believes that the extreme case scenario of the risk or hazards could metamorphose into a disaster with severe consequences. Even without such a realization, the experiences from other places give general insight to many people on the consequences of disasters or hazards. From this basis, Figure 3 summarizes the responses on whether disasters or COVID-19 are fatal, as witnessed across the world, Japan, and the Tokyo Metropolitan area. Looking at those who agreed with this fact, over 80% were affirmative for how both instances could be fatal to humans. This response set the motion for the other questions that followed, especially with a majority expressing a fear of disaster or infectious diseases such as COVID-19.

### 5.3. Disaster Experience 

**Figure 4:**
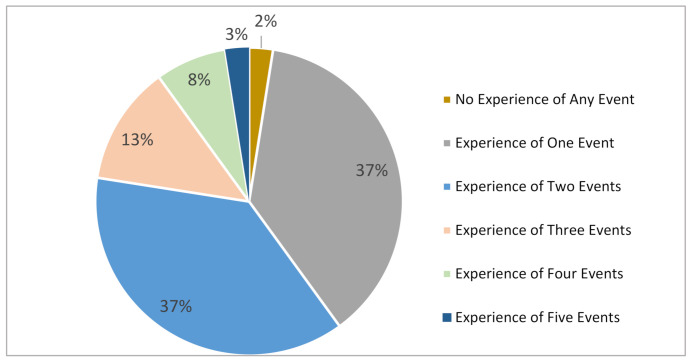
Disaster/hazard event experience.

Since there is a huge affirmation of the consequences of disasters and infectious diseases in the previous section, this section continues by revealing the answers from respondents if they had ever witnessed any such events in the city. From Figure 4, it is very clear that many had witnessed at least two events, with more than 40% having experienced more than four events in the city. These include earthquakes, typhoons, flooding, and others. These answers fall in line with the common knowledge that Japan, in general, is disaster-prone because only about 2% of its people are yet to experience or witness a disaster or hazardous event. This further compliment the earlier notion of disasters and it will be interesting to understand how the other results unfold in the next sections.

### 5.4. The Perspective of Future Occurrence of Disasters/Hazards

**Figure 5:**
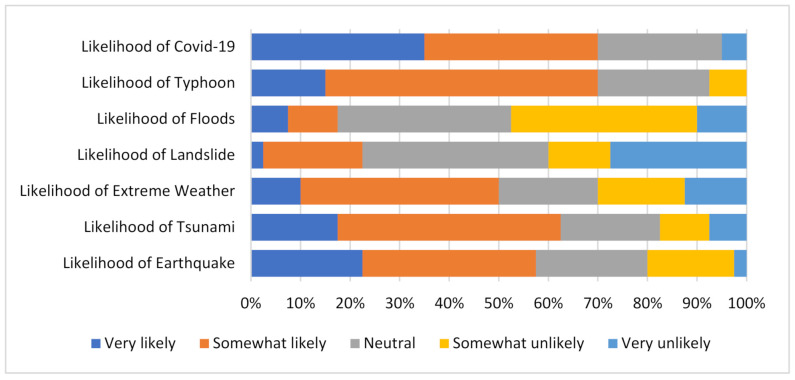
Perspective of the likelihood of events.

Disasters, infectious diseases, or hazards can be triggered by natural conditions or by human actions and this makes them unavoidable in many instances and inevitable in many others; however, to take any preventative action, one must be convinced that such events could happen. This is what Figure 5 depicts as the responses to the perception of the likelihood of some events occurring in the future. As shown in the figure, there are seven scenarios indicating the likelihood of certain events in the metropolitan area. Perhaps due to the current pandemic, most respondents asserted that there was a very high likelihood of a COVID-19 pandemic severely spreading in the Tokyo Metropolitan area. This is in line with the metropolis being the most populous with high numbers of infection rates in the country; however, when it comes to floods in the city, barely 20% believed that there was a likelihood that severe floods could occur, and with such question on typhoons, just as in the COVID-19 situation, there are over 70% of respondents with the view that a severe typhoon could likely occur in the city. 

The least held of the beliefs was for a landslide/mudslide. Per the distribution of risks in the metropolitan area, most landslides/mudslides likely occur in the mountainous western enclave of the metropolitan area; therefore, if people do not have the experience of living in those areas, there is the possibility that their perception of the likelihood of severe events of such nature occurring in the city would be lower. Consequently, from the responses, it was clear that many people have low expectations or beliefs about a landslide/mudslide occurring in the city. This notwithstanding, irrespective of where one lives in the city, it is the responsibility of a person to know the potential risks and hazards in the city should that person find him/herself in that area so that the appropriate counter-actions can be taken. Nevertheless, the results as a whole suggest there is positive thinking about the likelihood of many severe events in the city.

### 5.5. Efforts to Curtail the Challenges

**Figure 6:**
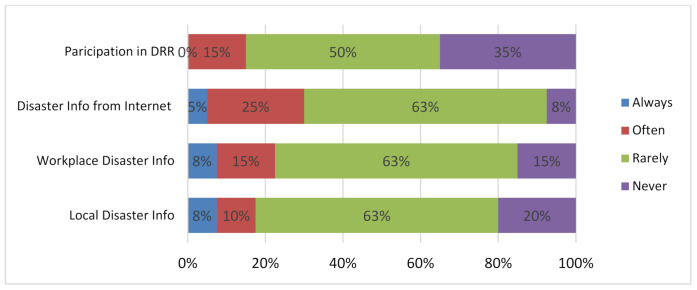
Efforts to curtailing challenges.

It is evidenced from the earlier sections that disasters or infectious diseases can be fatal because, per Figure 6, people have witnessed the occurrence of such events in the city, and when we looked at how such experiences were witnessed, it is found that the impact on respondents represented many aspects. The appropriate scenario would mean that people would take certain actions to avert similar situations in the future; hence the following responses gave clues to such initiations. As per Figure 6, there are mixed indications on the efforts to seek information on how to avoid, prevent or obtain knowledge on disaster risk reduction. When it comes to the participation in organized activities such as disaster drills or other communal disaster preparedness initiatives, it was seen that more than 80% of respondents barely take part in such activities; thus, only 25% could categorically state that they often took part in DRR activities. These characteristics further moves across other activities, such as obtaining information on disasters from the internet, from the workplace or from the school (for students in particular). Particularly at the workplace or with local information, many respondents at least make the effort to always check for such information, and although not as significantly as those who do the opposite. This was quite interesting as per the fact that, with all the awareness and the knowledge of the repercussions of disasters, fewer efforts have been put in place to seek updated and informed guidelines to reduce the potential risk or impacts should a mega-disaster occur.

### 5.6. Application of Risk Reduction Skills

**Figure 7:**
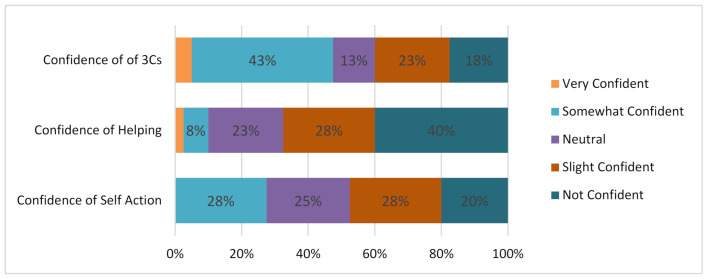
Confidence in recommended actions.

Knowing the presence of risk and potential disasters is very important but being sure and confident in what to do around such situations becomes the vital piece of the puzzle to ensure safety and resilience. From the questions that were posed to respondents on such issues, Figure 7 shows very little indication of a high confidence in undertaking certain measures, especially with answers such as “somewhat confident”, which was the dominant response to the question, “how confident are you that you are avoiding the 3Cs of COVID-19 infections (e.g., closed spaces, crowded spaces, close-contact settings)?”. Although this casts doubts on the confidence level in preventing infectious disease, the responses to the subsequent measures are quite concerning. For instance, by operationalizing the self-help and mutual assistance initiative by the local government authorities, being able to help oneself and others is the core or major element for successful community resilience, but the respondents with no confidence at all dominated as to whether they can take action themselves or if they can even help others in emergency situations; however, the confidence for helping themselves was higher than that for helping others.

### 5.7. Perceived Threat Severity and Susceptibility

**Figure 8:**
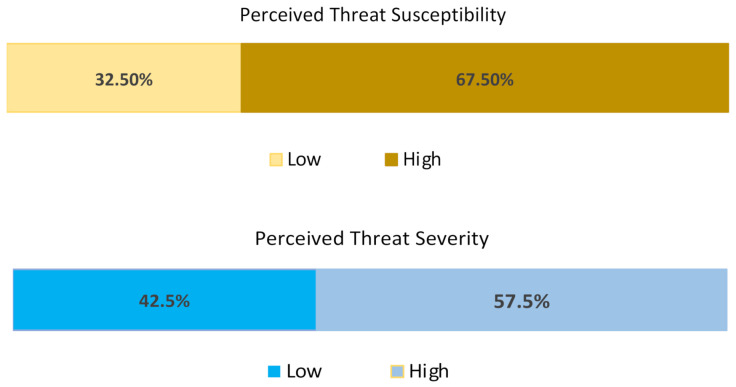
Perceived threat severity and susceptibility.

Perceived threat severity and susceptibility are the two components that make up the general perspective of how people view threats as a whole. Using mean values of the variable under the perceived threat severity, a mean of the total threat severity was calculated as 4; hence, all values below the mean values represented a low threat severity, while values above the mean value equaled a high perceived threat severity. Based on this assumption, the perception of threat severity in the metropolis is nearly 60%, as shown in Figure 8. This indicates that people believed in threats hazards in the city and assumed that disasters could cause severe devastation. 

Utilizing the mean scores of variables representing the likelihood of events, a mean value of the total was calculated to be 3; hence, values below this mean figure were categorized under a low threat susceptibility while a high threat susceptibility was when the values were higher than the mean value. From Figure 8 again, the perceived threat susceptibility was nearly 70%. This suggests that some foreign residents in the city have high perceptions when it comes to the likelihood of severe hazards or disasters occurring, but that such a belief outweighs how severe they envisage the threat itself.

### 5.8. Perceived Self and Response Efficacies

**Figure 9:**
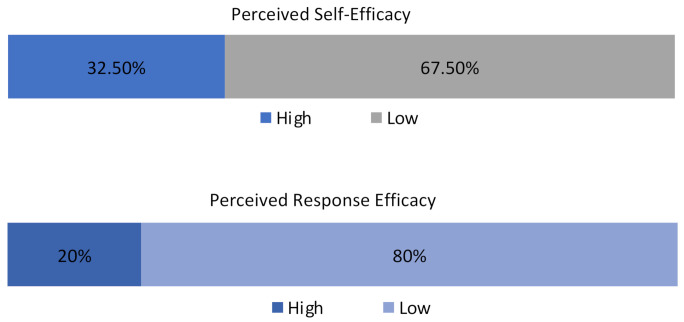
Perceived self and response efficacies.

The components of general efficacy are made up of two variables, namely, a perceived self-efficacy and response efficacy. Similar to other calculations, the mean total of the variables that make up the confidence of people to undertake certain recommended measures are constituted. By taking the mean of these values, a low perceived self-efficacy is deduced by values lower than the mean figure. For this purpose, the mean value was estimated to be 3; therefore, Figure 9 shows that 67.5% of respondents have low self-efficacy with only a few possessing the confidence to handle many challenges should disasters occur. As discussed in the earlier sections, this low self-efficacy stems from how some foreign residents do not have the confidence to help others, observe the COVID-19 3Cs, etc.

Furthermore, the figure shows the composite variables of how some foreign residents attempt to seek information on how to improve their resilience toward disasters or hazards in the city. These are the variables for the perceived response efficacy. The mean values that were estimated from the mean values of the variables to be 2, and values below this figure, were classified under a low perceived response efficacy, while a high perceived response efficacy was estimated as being values higher than the mean value. From Figure 9 again, the perceived response of the respondents is as low; representing 80%. Meaning that their attitude toward seeking information and other important knowledge on disasters is low. This may be a contributing factor to the low self-efficacy of some foreign residents because if no new information is sought, then the ability to respond to or be confident in the actions to take becomes questionable, as seen from these survey results.

### 5.9. Attitude towards Disaster Preparedness 

**Figure 10:**
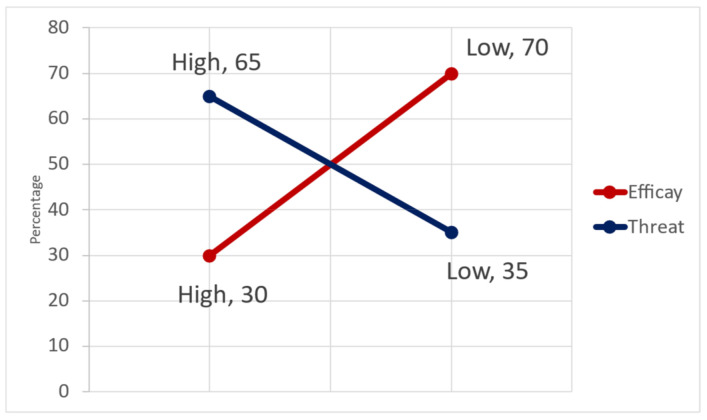
Attitude on disaster preparedness.

Several elements contribute to how people react to disasters and other emergencies. As stated earlier, the EPPM model proposes one perspective to understand such behaviors, and the preceding sections have laid out the foundation to the understanding of some foreign residents to this process. In this section, all the variables that describe how threats and efficacies are perceived toward disaster preparedness are estimated. By taking the mean value of the sum of the perceived threat susceptibility and severity, all the figures lower than the mean value of 7 were classified as a low threat perception, while the high perceived threats were determined by classifying all the values greater than the mean value; hence, Figure 10 shows that the perceived threat is high at 65%. This means that the information dissemination by city governments may have gained enough recognition in terms of its threat message in the sense that that disaster and hazardous conditions pose to lives and properties in the city. Per this assumption, the EPPM stipulates that people will then assess their capacities to curtail these threats, since there is a general recognition of the threats in the city.

Therefore, by utilizing the variables of response and self-efficacies, the capacities of some foreign residents towards efforts against the threats were assessed by taking the mean score of the sum of both efficacies. With a mean score of 5, the level of capabilities of the foreign residents in the form of efficacies was estimated by categorizing all the values lower than the mean score as a low efficacy toward threats. High efficacies were further estimated by categorizing all the values greater than the mean score. From these processes, Figure 10 further shows that only 30% of some foreign residents have high efficacies. The remaining 70% have the perception that they are unable to undertake some recommended or existing measures required to enhance the resilience capacities toward disaster preparedness. Per the assumption of the EPPM, some foreign residents are refraining from receiving more information about disasters because they feel they are not in the position to undertake the recommended measures; therefore, in reference to the assumptions of EPPM, they are rather controlling their fear. Fear control means that people will not move to the next stage of taking any action for disaster preparedness.

## 6. Discussion 

Learning from previous studies and the realization of a new direction for characterizing the impediments to disaster preparedness of foreign residents in the metropolitan area, a quick overview can be drawn from the broader notion of whether awareness itself leads to actions. The authors of a previous study [34] discuss awareness as part of a social change marketing strategy in which a “good idea” is “sold” to people with the intention to change their attitudes or actions; however, they point out that there are different stages in this changing process and caution that every awareness strategy must endeavor to acknowledge this to be able to provide the required information. For this purpose, many refer to the process as the “awareness campaigns” [35]. For instance, the World Health Organization (WHO) has several health awareness campaigns including: World No Tobacco Day (WNTD), World Malaria Day (WMD), World Immunization Week, the World Hepatitis Day, and many more [36]. Similar to these causes are numerous campaigns aimed at disaster prevention and risk reduction which are observed across the world. An example includes the International Day for Disaster Risk Reduction observed every October 13 as a measure to promote risk reduction activities, and in Japan, such campaigns include the Disaster Response Volunteer Day, Disaster Prevention Day, Tsunami Preparedness Day, and many others [4]. 

In an article written under the ‘Essentials of Social Innovation’, Christiano and Neimand 2017 outline and provide evidence to suggest that awareness campaigns can fail, can reach the wrong audience, can lead to a backlash, or can even create harm [37]; however, successful awareness campaigns can also create the needed knowledge originally unknown to others. Juxtaposing these two instances to this research, awareness of disasters in the form of contact engagements, online/virtual information dissemination, disaster prevention pamphlets, and booklets used by the Tokyo Metropolitan Government, seems to have achieved some success in terms of the characteristics of foreign residents as shown from the results. Referencing Section 5.2, Section 5.3 and Section 5.4 in this study shows that some foreign residents are indeed aware or have knowledge about disasters and risks in general; thus, there is a high level of risk awareness not only with one hazard type but with many other hazards either within the Tokyo Metropolitan area or in Japan as a whole. The realization of risk as a matter of disaster prevention and management is an important element for community-level initiatives because such awareness has the ability to galvanize, mobilize and encourage voluntary actions toward risk reduction [38]. Empirical evidence shows that a community’s awareness of risk reduces the disaster impacts and changes its lifestyles and perceptions [38]. This notwithstanding, the evacuation behavior during the 2018 Sulawesi Earthquake Tsunami in Indonesia gives a vivid example of the fact that risk awareness does not always lead to taking action during the event of a disaster [39]. These scenarios highlight the relevance of the awareness levels of some foreign residents as depicted in this study to pose the question as to whether the knowledge of a hazard availability can contribute to taking action to prepare for disasters; thereby, being able to help themselves and others. This has become imperative because the revised Disaster Countermeasures Basic Law 2013 of Japan mandates all local governments in the country to prepare lists of people who would need assistance in disaster situations. These include people with a disability, people in nursing homes, and others. Although it is envisaged that these groups of people would be evacuated by trained agencies and other volunteers, the case of mutual help activities during the 6.9 magnitude Great Hanshin-Awaji Earthquake in 1995 and the 9.0 magnitude Great East Earthquake in 2011 show that much of these works would be undertaken by residents close to those vulnerable people. However, the ability to envisage such perceived actions in certain circumstances is often understood through behavioral models which, until recently, had seen little application in the field of disaster management. 

Utilizing one of these behavioral models, such as the EPPM in this study, reveals that if a disaster is to occur, the confidence of foreign residents to undertake some recommended countermeasures is rather lacking and this is manifested in how most are hesitant to be involved in disaster preparedness activities such as partaking in organized activities, searching for risk information and others (see Figure 7). This stage is what is referred to as “fear control” [22]. Juxtaposing these issues brings to light the element of self-efficacies which, per the existing studies, could be an important component to potentially impede disaster preparedness [28] because a “person’s self-efficacy is a strong determinant of their effort, persistence, strategizing, as well as their subsequent training and performance” [40]. As the survey results indicate, with the low perceived self and response efficacies of some foreign residents in the Tokyo Metropolitan area, a matter arises regarding improvements in the efficacies in disaster preparedness.

Improving efficacies have, however, been theorized and implemented in previous studies through four main indicators, such as through enactive mastery experiences, vicarious experiences, verbal persuasion, emotional and physiological states, and mastery experiences [35,36]. Enactive mastery experiences boost efficacies when there is an attempt at something similar to successful tasks taken from previous experiences. A vicarious experience is when a person improves his/her efficacies by observing the success of an action by a person with similar qualities. A verbal persuasion is, rather, a personal approach where people are encouraged to undertake certain tasks with the belief that they would succeed [41,42]. The emotional and physiological states of a person, on the other hand, determine the belief in the likelihood to be successful in the tasks [34,35]. These are all prerequisite elements in community and self-preparedness [28,39,43]. To converge all the issues along with the approach to improving efficacies in the case of foreign residents in the Tokyo area, previous research highlights the relevance of enhanced public engagement strategies as an integration mechanism for foreign residents in disaster preparedness. It emphasizes that the system of engagement of foreign residents in disaster preparedness activities in the Tokyo metropolitan areas comprises both virtual and physical environments which aim to inform, consult, involve, and promote collaborations among all parties [29]. These descriptions are in line with the properties of self-efficacy improvements [34,35,36]; therefore, by utilizing the public engagement aspect of the virtual and physical environments, the elements of self-efficacy improvement could be aligned as such. Studies have shown that verbal persuasion, vicarious and inactive experiences are best initiated through physical interactions [44] and the manifestation of such in disaster prevention has been visible in the disaster evacuation process [45]. 

**Figure 11:**
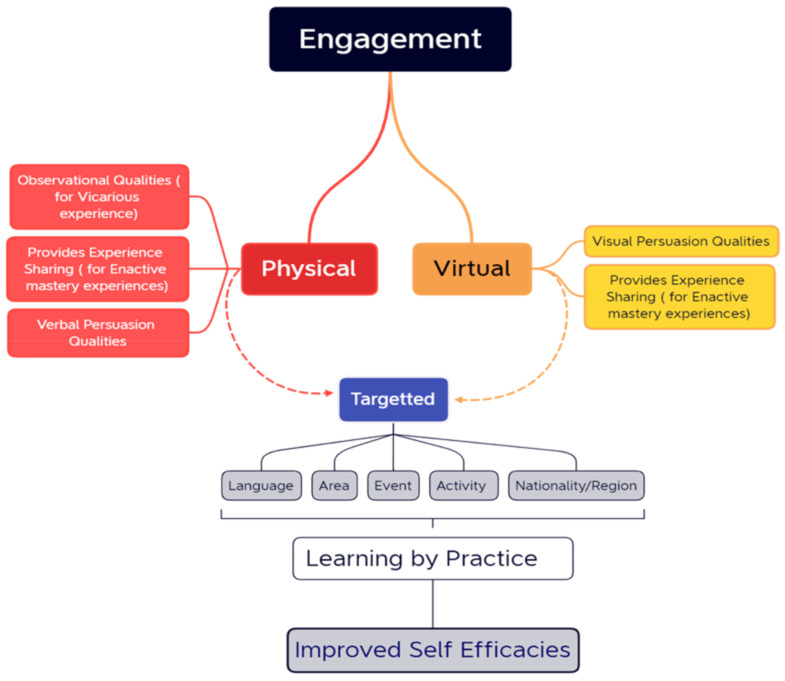
Proposed framework of efficacy improvement.

Furthermore, the COVID-19 pandemic brought to light the capacities of modern technology and innovations in bridging distances and space, such that it could further be incorporated into activities to enhance the efficacies. Implementing such approaches means that in the Tokyo metropolitan area, information in the form of engagements could be customized based on the characteristics of foreign residents in terms of their Japanese language level, where they live in the city, the type of activity familiar to them, and other qualities, so that people could learn and improve their efficacies based on the learning experience. Evidence from the existing studies shows the relevance of targeted information dissemination in disaster preparedness [32]. A suggested framework utilizing such scenarios has been proposed in Figure 11. 

## 7. Key Findings and Conclusions

The study sought to understand the issues of disaster preparedness of foreign residents from their psychological perspective, where the decision to undertake disaster preparedness activities stems from a series of decision-making processes. The following summarizes the output and concluding remarks of the issues identified.

The Perception of risk is a factor of perceived threats: awareness was tested on how foreign residents perceive risks, and it was established from a conducted survey that indeed the perception of risk do not differ from the viewpoint of its threats. Using both infectious diseases and disasters as yardsticks, there was an overwhelming recognition that both instances could cause fatalities, with about 80% of respondents agreeing to this fact. This further translated in to how they perceive the likelihood of an extreme event in the future; therefore, by applying an EPPM model, nearly 58% of the foreign residents the perception of high threat severity in the metropolis, while others have high perceptions when it comes to the likelihood of severe hazards or disasters occurring with about 67% of perceive the susceptibility score . In general terms, 65% perceived earthquakes, extreme weather, landslide/mudslides, typhoons, and floodings as threats in the metropolis.

Low disaster prevention response capacities: although the above results showed high threat perceptions, the capacities to undertake recommended countermeasures by foreign residents are identified as low; thus, considering the abilities to help themselves or others, less than 10% of the respondents could confidently say they have the abilities and confidence to help others in the event of disasters. Furthermore, less than 30% indicated that they have the confidence to help themselves and would be able to follow every protocol in disaster events. This was further realized in the limited willingness attitude of some foreign residents to seek disaster preparedness information from their workplace, school, local communities or on the internet.

Fear control attitude of foreign residents to disaster preparedness: the study reveals that the representation of disasters and other hazards as threats have heavily impacted the perception of threats that they posed, with a 65% indication of a high threat as opposed to a 35% indication of low perceived threats. Upon the countermeasure recommendations that come with risk information, there is about 70% of people with low efficacies towards such threats with only 30% having high efficacies. The EPPM model explains this scenario as that, because the perception toward risk or threat severity and susceptibility is high, much of such information is received; however, upon assessing their response capacities and their abilities to perform the recommended countermeasures, the respondents would turn to ignoring further messages on countermeasures because they don’t have the abilities not undertake them, thus, suppressing their fear by performing what is called “fear control”. A population in fear control refuses to proceed to take actions toward risk management; therefore, in this case, it suggests the reason why there is low attitude towards seeking disaster prevention information, partaking in disaster drill exercises, and other recommended disaster prevention protocols as previously studied. To improve the situation, a framework of efficacy improvement has been proposed as a continuous learning process that serves as an information dissemination platform as well as a mechanism for the integration of foreign residents into the communities in the city. The relevancy of such continuity brings forth the different dimensions of the type of risk and the duration. While earthquakes and other disasters have short reaction times, the COVID-19 pandemic has demonstrated the need to sustain risk awareness and prevention momentum. It is with this idea that a targeted engagement activities are vital.

To understand which other targeted actions should be put in place to enhance the risk awareness and disaster preparedness on the part of both foreign and Japanese resident populations, future research could investigate the role of big data and other emerging technologies in analyzing behavioral differences toward disasters.

## 8. Limitations

Although the research gives an insight into the disaster preparedness perception of foreign residents in the Tokyo Metropolitan area, there are some limitations. The first is the sample size. This study is limited in terms of the number of responses from the survey which further limits the generalization of the results to the entire foreign residents’ population in the metropolis. Further, the questionnaire for the survey was designed in the English language which may have excluded some respondents with a low English proficiency. Future studies should consider such elements to broaden the research matter.

## Data Availability

A copy of the questionnaire for this study is available from the corresponding author upon well-founded request.
